# Supplementary material for: Patterns of Intron Gain and Loss in Fungi
Source: PLoS Biol. 2004 Nov 30;2(12):e422. doi: 10.1371/journal.pbio.0020422 (PMC532390; doi:10.1371/journal.pbio.0020422)
Supplement: Table S1 — Also available at http://genes.mit.edu/NielsenEtAl/. (4.3 MB ZIP). [file pbio.0020422.st001.zip › NielsenEtAl/html/204.html]

AN4412.1.NCU03363.1.MG03931.1.FG01656.1


```
 CLUSTAL W (1.82) Multiple Sequence Alignments - Introns Inserted


Sequence 1: NCU03363.1	1106 aa
Sequence 2: FG01656.1	1094 aa
Sequence 3: MG03931.1	1102 aa
Sequence 4: AN4412.1	1073 aa
Alignment Length: 1125 aa
Number Identitical Residues: 536 aa
Alignment Score (without introns) 25953


MG03931.1 	MADLFDVFDEQPEAPAVGLDRNSSDTKTKKTKKSKRDKSKDKSKKRKADGHVKNLGAQDN
NCU03363.1	MDDLFEVFEEQPRAQKKRKASPDVEMADGAAAAAPVANAAAAVTHPQAQAAPAPQKDNID
FG01656.1 	MDELFDVFEG------------NVEAPSASEDESRQNRKGDKTKKRKANAINGKHGDADK
AN4412.1  	MDELFDVFED---------------KPQAAQLSEPRRPKKEKSKKRQINGDVKENGENAK
          	* :**:**:                                  .: : :.         .

MG03931.1 	GAEAQPDPQEDVEMAN-----GTQPSTGDAGSEESSSDEHQDIKRRKKEGEADPILTDTF
NCU03363.1	NNDNNNNNTTTSAVQPTIPNGDAAAAADDVQSPQSPHG-LGDNKRRKKTDEAEPIMTDAF
FG01656.1 	SEDVDMDNNTTEESK-------DEASSDDESSEESAS--QQDAKRRKKEEAVGPVITDTF
AN4412.1  	PKENIAPDVAEQENK-------------EEKSSGSDNNNQPETKRLRLEEEPEPVVADLF
          	  :                         :  *  *  ..  : ** :      *:::* *

MG03931.1 	ETAQSRQVAAAEGFGTKEED-SLVLSHNIQHQVALPPDLDYVYVPLSEHKPPEEPARKYA
NCU03363.1	QTAESREVTGAQGFAPTEGE-SIVLSHNIQHQVALPPDLDYEYIPLSEHKPPAEPARTYS
FG01656.1 	QTAESREVAGAATFTEQDS--SLVLSHNIQHQVALPPDLDYEYVPLSEHKAPEQPARTWN
AN4412.1  	ETAQEREIAGSAGLQAEKEAGPVVLSHQVRHQVAIPP--KYPYVPISQHKPPENPARVWP
          	:**:.*:::.:  :   .  ..:****:::****:**  .* *:*:*:**.* :*** : 

MG03931.1 	FKLDPFQSISVASIERDESVLVSAHTSAGKTVVAEYAIAHCLKRNQRVIYTSPIKALSNQ
NCU03363.1	FKLDPFQALSVASIEREESVLVSAHTSAGKTVVAEYAIAQCLKKNQRVIYTSPIKALSNQ
FG01656.1 	FKLDPFQSLSVASIEREESILVSAHTSAGKTVVAEYAVAQCLKRNQRVIYTSPIKALSNQ
AN4412.1  	FTLDPFQQVAVSSIQRGESVLVSAHTSAGKTVVAEYAIAQSLKNNQRVIYTSPIKALSNQ
          	*.***** ::*:**:* **:*****************:*:.**.****************

MG03931.1 	KFRDFQAEFGDVGLMTGDVTINPTASCLVMTTEILRSMLYRGSEITREVGWVVFDEIHYM
NCU03363.1	KYRDFQAEFGDVGLMTGDVTINPTASCLVMTTEILRSMLYRGSEIMREVAWVVFDEIHYM
FG01656.1 	KYRDFEAIFGDVGLMTGDVTINPTASCLVMTTEILRSMLYRGSEIMREVAWVVFDEIHYM
AN4412.1  	KYREFAAEFGDVGLMTGDVTINPTATCLVMTTEILRSMLYRGSEIMREVAWVVFDEIHYM
          	*:*:* * *****************:******************* ***.**********

MG03931.1 	RDAT~RGVVWEETIIMLPDKVRYVFLSATIPNAFQFAEWIAKIHRQACHVVYTDFRPTPL
NCU03363.1	RDKI~RGVVWEETIILLPDKVRYVFLSATIPNAFQFAEWIAKIHRQACHVVYTDFRPTPL
FG01656.1 	RDKT~RGVVWEETIIMLPDKVRYVFLSATIPNAFQFAEWIAKIHHQACHVVYTDFRPTPL
AN4412.1  	RDAT1RGVVWEETIILLPDKVRYVFLSATIPNAMQFAEWITKMHNQPCHVVYTDFRPTPL
          	**   **********:*****************:******:*:*.*.*************

MG03931.1 	QNYVFPAGGDGIYIMVDEKGVFREKKFMEAIGSIAGKN--DDDDSMPRQKGKGKNKKAVK
NCU03363.1	QNYFFPAGGKGILLIVDEKGNFKENNFNQAMAMIEEKKGTDSNDWSAKQKGKGKNKKTNK
FG01656.1 	QNYFFPAGGSGARLIVDEKSNFNEQNFNKVMQEVEEKKGADPNDPNARQKGKGKNKKTNK
AN4412.1  	QHYFFPAGSEGMHLIVDEKGVFREENFQKAMSSIADKKGDDPADALAKRKGKGKDKKLNK
          	*:*.****..*  ::****. *.*::* :.:  :  *:. *  *  .::*****:**  *

MG03931.1 	NT-VPDSKADITKIIKMIMRRAYHPVIVFNFSKRECENLALKVSTLNFNHESEQKLVEDI
NCU03363.1	GGEAADEKADIAKIIKMILKKNFQPVIVFNFSKRECEQMALASSSMKFNAPDEENMVNKV
FG01656.1 	GG--ADNGSDIAKIIRMTIKKKFNPVIVFNFSKRECENMAMNISSLSFNDDSEKAMVRKV
AN4412.1  	GG--TQEKDDIYKIVKMIMLKSLNPVIVFSFSKRECEFYALKMKSLAFNDDSEKEMVSKV
          	.   .:.  ** **::* : :  :*****.*******  *:  .:: **  .*: :* .:

MG03931.1 	FRNAIMSLSEEDQGLPQIQHLLPLLKKGIGVHHSGLLPILKETIEILFQENLIKVLFATE
NCU03363.1	FENALASLSEDDKNLPQISNILPLLRKGIGVHHSGLLPILKETIEILFQEGLIKVLFATE
FG01656.1 	FHNAIESLSEQDRELPQIINLLPLLERGIGVHHSGLLPILKETIEILFQESLIKVLFATE
AN4412.1  	FNSAIEMLSEEDRNLPQIQNILPLLRRGIGVHHSGLLPILKETIEILFQEGLIKVLFATE
          	*..*:  ***:*: **** ::****.:***********************.*********

MG03931.1 	TFSIGLNMPAKTVVFSQVKKFDGVKERPLTPSEYIQMSGRAGRRGLDDRGIVIMMIDDKL
NCU03363.1	TFSIGLNMPARTVVFTQVTKWDGQQRRPLTSSEYIQMAGRAGRRGLDDRGIVIMMVDDKL
FG01656.1 	TFSIGLNMPAKTVVFTQVTKWDGVKRRPLTSSEYIQMAGRAGRRGLDARGIVIMMIDDKL
AN4412.1  	TFSIGLNMPAKTVVFTSVRKFDGFSQRWVTPSEFVQMSGRAGRRGLDDRGIVIMMIGEEM
          	**********:****:.* *:** ..* :*.**::**:********* *******:.:::

MG03931.1 	DPETAKGMVCGQQDRLNSAFHLGYNMILNLQRIETVSPQYMLERCFYQFQNASSVPALEK
NCU03363.1	EPETARAIVVGNQDKLNSAFHLGYNMVLNLLRIEAISPEYMLERCFFQFQNAASVPQLER
FG01656.1 	EPDTAKEIVTGHQDRLNSAFYLGYNMILNLLRIEAISPEFMLERCFHQFQNAASVPSLEK
AN4412.1  	DPAVAKEIVRGEQDRLNSAFHLGYNMILNLMRVEGISPEFMLERCFYQFQNTAGLAGLEK
          	:* .*: :* *.**:*****:*****:*** *:* :**::******.****::.:. **:

MG03931.1 	K~LIELQQQRDAMVIPDESTVKDYHTVRQQIEAYKKDMEAVIQHPNNCLEFMQPGRLIRI
NCU03363.1	E~LISLQQERDAIIIPDESIVKDYYGVRQQLEEYNKDMVFVIQHPQNCLGFFQEGRLIHI
FG01656.1 	E~LMSLQQERDSTSIADESTVKDYYQIRQQLSAYTRDMRTVIQHPNYSISYLQPGRLVQI
AN4412.1  	E1LAELEEKRANMTISDEGTIREYYDIRTQIDQFNDDVRAVISHPEYSVPFLTPGRLLHI
          	: * .*:::*    *.**. :::*: :* *:. :. *:  **.**: .: ::  ***::*

MG03931.1 	KTP---------DGVDFDWGVVVNFLPRKPVKFGEEEPPPQESYLMDVALFVSEDSISPP
NCU03363.1	KSP---------SGVDYGWGVLIKHIQRQTPKNGQPPYPEQESYVLDVLLKVS-----GD
FG01656.1 	YNPKDKNESIAGNGTDFGWGVIVNQTPRRAPKLNEPEYIPQEAHVIDVLLPISRS--SAD
AN4412.1  	KYK----------DFDFGWGVVVNIKKRKPQKNSE-ELTGHASYIVDVLLRVADG-----
          	             . *:.***:::   *:. * .:     : ::::** * :: .     

MG03931.1 	PHASVSKEGHIADGVEPSGSVD-HGRLEVVPCLLTCMVAISQIRIFMPKD--MDHLGKSQ
NCU03363.1	FNPKTRGEGPMPEGIMPAGKDSKNARWEVVPCLLNCLRALGQLRVFLPKR-LESADEKDG
FG01656.1 	FHPGHPAE-EMPPGIKPCNDDD-DIKFAVVPCLLTCIKAISQIRLFLPKEGLKSDSDKDT
AN4412.1  	SSSGTKTFEDLPQGVRPPKEGE-KSQMEVVPLLLNCIQAISHVRMIVPKD-LQSKDSRTD
          	  .       :. *: *  . . . :  *** **.*: *:.::*:::**    .   :  

MG03931.1 	VSHGLEEVQRRFPDGVPILDPIENMGIKDDSFKKLMR~KIEVLESRLLSNPLHGSPNLPE
NCU03363.1	VGKAVDEISRRFPDGIPILDPMENMGINDDSFKKLLR~KIEVLESRLVANPLHNSPLLVE
FG01656.1 	LTKSLMEVKRRFPDGLPVLDPIENMEITDDSFKKLLR~KIEVLESRLLANPLHLSPLLPS
AN4412.1  	MGKKVEQIKKRFPDGIAVLDPIEDMGIKDDEFKKTLR0KIEVLESRLVTNPLHNSPRLEE
          	: : : ::.:*****:.:***:*:* *.**.*** :* *********::**** ** * .

MG03931.1 	LWDKYKIKTELTSQIKETKRAINKSYSIAQLDELKSRMRVLRRLNFINENEVVQMKARVA
NCU03363.1	LWNQYSLKMQLGEQIKEKKKAIARAHSVAQLDELKSRKRVLRRLGFINDAEVVQMKARVA
FG01656.1 	LWDQYHAKVKLTEKVKETKKSIAKAYSIAQMDELKSRKRVLRRLGFINDAEVVQLKARVA
AN4412.1  	LYEQYAEKLDLGNKIKATKKKISEGMAIQQLDELKCRKRVLRRFGFINEAEVVQLKARVA
          	*:::*  * .* .::* .*: * .. :: *:****.* *****:.***: ****:*****

MG03931.1 	CEISSTEGHELVISELLFNGFFNELEPEVCAAILSCFVFDEKMEGTP--LKEDLDKLVRE
NCU03363.1	CEISSTEGHELLLAELLFNRFFNELSPEICACILSCFIFDEKIETQA--LKEELAKPFRE
FG01656.1 	CEVSSTEGHELLLSELLFDRFFNEQTPEMCAAVMSCFIFDEKVEAPA--LKEELQKPYRE
AN4412.1  	CEIST--GDELMLSELLFNGFFNKLTPEQAAAVLSVFVFEEKTKETPPLSKEELAKPLKE
          	**:*:  *.**:::****: ***:  ** .*.::* *:*:** :  ..  **:* *  :*

MG03931.1 	IHAQAKTIARISRESKLDVSDEQVVGNLKWQLTETVLAWAKGRPFAEIC2KMTNAYEGSL
NCU03363.1	IQAQARIIAKVSAESKLDVNEDEYVQSLKWQLMETVLAWAQGRPFSEIC2KMTNVYEGSL
FG01656.1 	IQAKARIIAKVSQECKLDVNEEEYAQKLKWQLMETVYTWAQGRPFVEIC2KMTNVYEGSL
AN4412.1  	IQAQARIIAKVAQESKLAVSEEDYVQSFHWELMEVIYEWANGKSFADIC2GMTDVYEGSL
          	*:*:*: **::: *.** *.::: . .::*:* *.:  **:*:.* :**  **:.*****

MG03931.1 	VRIMRRLEELLRQMAEAGKVMGSEMLQKKFEKALSLISRDVVSAASLYL
NCU03363.1	IRLFRRLEELLRQMAEAARVMGSEELKDKFELSLSKIRRDIVSFNSLYL
FG01656.1 	IRLFRRLEELLRQMAQAAKVMGNEDLTKKFEESLSKIRRDIVAAQSLYL
AN4412.1  	IRVFRRLEECLRQMAQAAKVMGSEELESKFETALTKVRRDIVAAQSLYL
          	:*::***** *****:*.:***.* * .*** :*: : **:*:  ****
```
